# *VEGF* and *bFGF* gene polymorphisms in Polish patients with B-CLL

**DOI:** 10.1007/s12032-013-0456-4

**Published:** 2013-01-19

**Authors:** Tomasz Wróbel, Grzegorz Mazur, Justyna Dzietczenia, Katarzyna Gebura, Kazimierz Kuliczkowski, Katarzyna Bogunia-Kubik

**Affiliations:** 1 Department of Haematology, Blood Neoplasms and Bone Marrow Transplantation, Wroclaw Medical University, Wybrzeze L. Pasteura 4, 50-367 Wroclaw, Poland; 2 L. Hirszfeld Institute of Immunology and Experimental Therapy, Polish Academy of Sciences, R. Weigla 12, 53-114 Wroclaw, Poland; 3 Department of Internal, Occupational Diseases and Hypertension, Wroclaw Medical University, Borowska 213, 50-556 Wroclaw, Poland

**Keywords:** B cell chronic lymphocytic leukemia, *VEGF* polymorphism, *bFGF* polymorphism, Disease progression

## Abstract

Among a variety of angiogenic factors involved in the B cell chronic lymphocytic leukemia (B-CLL), vascular endothelial growth factor (VEGF) and basic fibroblast growth factor (bFGF) were identified. Their levels have been regarded as prognostic markers of the progression of disease. The objective of the present study was to assess whether polymorphisms located within the genes coding for these key angiogenic activators contribute to disease susceptibility and/or progression in patients with B-CLL. For this purpose, 180 individuals were investigated, including 68 B-CLL patients and 112 healthy controls. All individuals were typed for the *VEGF (936 C* > *T)* and *bFGF (*−*921 C* > *G)* alleles using PCR–RFLP technique. Only a slight prevalence of the *VEGF T* variant was observed among patients as compared to healthy individuals (*p* = 0.095) with a significant difference when high risk (stage III/IV) patients were considered (OR = 3.81, *p* = 0.045). No other significant association was observed between the *VEGF* polymorphism and progression of the disease. The *VEGF* alleles and genotypes segregated similarly in patients with different stage of the disease according to Rai classification. No significant relationships were also observed for the *bFGF* polymorphism with either susceptibility to B-CLL (when compared to control group) or progression of the disease. These results suggest the possible association of the *VEGF* polymorphism with high risk B-CLL.

## Introduction

Dysregulation of angiogenesis occurs in various pathologies and is one of the hallmarks for cancer. The importance of this biological process in normal hematopoietic cell development and the pathophysiology of several malignancies, including B cell chronic lymphocytic leukemia (B-CLL), has been recently reported [1–3]. Patients with CLL have been demonstrated to have detectable levels of both plasma and cellular pro- and anti-angiogenic cytokines, as well as abnormal neovascularization in the marrow and lymph nodes [4–6]. Recent evidence suggests that vascular endothelial growth factor (VEGF)-based autocrine pathway promotes the survival of CLL B cells in part through upregulation of anti-apoptotic proteins [7]. Moreover, interactions between CLL B cells and their microenvironment generate alterations in the secretion of angiogenic factors that result in enhanced leukemic B cell resistance to apoptotic cell death [8]. Among a variety of angiogenic factors involved in the CLL, vascular endothelial growth factor and basic fibroblast growth factor (bFGF) were identified [9]. Their levels have been regarded as prognostic markers of the progression of the disease [10–14] in patients with B-CLL, including those of Polish origin. The data on the role of the VEGF and bFGF in CLL are summarized in Table 1.

**Table 1:** Current status of data on the role of VEGF and bFGF in CLL

Factor	Observation/impact on the disease	Reference
VEGF	VEGF is expressed differently in plasma and serum of CLL patients	[1]
	VEGF mRNA and protein are produced in CLL cells	[4]
	VEGF is expressed on B-CLL granulocytes and lymphocytes; VEGF receptors, VEGFR-1 and VEGFR-2, are expressed on B-CLL cells	[6]
	VEGF levels do not differ between plasma of CLL patients and healthy controls	[5]
	Bone marrow stromal cells (BMSC) treated with CLL microvesicles produce VEGF on a higher level than untreated healthy BMSC, but not as high as CLL-BMSC;	[3]
	VEGF_165_ is the main and overexpressed isoform in CLL-BMSC compared to healthy cells;	
	VEGF_121_ is poorly expressed, and isoforms VEGF_189_ and VEGF_206_ are not detected	
	VEGF serum level is higher in CLL patients than in healthy individuals;	[13]
	VEGF and VEGFR-2 levels are significantly higher in serum of patients in III or IV than in those in 0–II Rai stage of the disease;	
	VEGF and VEGFR-2 serum levels correlate in CLL patients	
	VEGF supports antiapoptotic and cytoproliferative effect of CD154 in CLL cells;	[7]
	Inhibition of VEGF and its receptor decreases CLL cells survival	
	VEGF produced by bone marrow stromal cells, but not by CLL cells, decreases CLL cells apoptosis	[8]
	Increased expression of VEGF receptors correlates with clinical stage	[24]
	High serum levels of VEGF correlate with increased risk of disease progression in early B-CLL	[11]
	Low level of VEGF correlates with worse outcome in B-CLL patients with low level of β2-microglobulin (good prognosis indicator) what may lead to decreased survival of patients in 0 to II stadium of the disease	[12]
	Targeting VEGF receptors effectively induces apoptosis in primary CLL cells and reduces tumor growth in a VEGF-positive CLL-like xenograft mouse model	[25]
bFGF	Plasma levels of bFGF in CLL patients are significantly higher compared to levels of other proangiogenic molecules (i.e. VEGF) in plasma of these patients	[1]
	Plasma levels of bFGF are significantly higher in patients with B-CLL compared to healthy controls	[5]
	Serum levels of bFGF are statistically higher in patients with B-CLL than in healthy controls; serum bFGF level was significantly higher in patients with progressive than in those with stable disease	[14]
	bFGF upregulates BCL-2 expression in B-CLL	[9]
	Increased expression of bFGF correlates with clinical stage	[10]

The objective of the present study was to assess whether polymorphisms located within the genes coding for these key angiogenic activators (VEGF and bFGF), contribute to disease susceptibility and/or progression in patients with B-CLL.

## Materials and methods

### Patients and controls

Sixty-eight patients (F/M = 27/41), aged 39–85 (median 69) years, with B-CLL were investigated. B-CLL was diagnosed according to defined clinical, morphological and immunological criteria. All patients gave their informed consent prior to their inclusion in the study. The study has been approved by the appropriate ethics committee.

Patients were treated at the Department of Hematology, Wroclaw Medical University. According to the modified Rai classification [15], there were 17, 28 and 12 patients in stage 0, I and II of the disease, respectively. The other 11 patients presented with more advanced disease: 6 and 5 with stage III and IV, respectively. In addition, 112 healthy individuals of both sexes (F/M = 57/55) served as a control group.

### *VEGF* and *bFGF* genotyping

DNA was isolated from the whole blood taken on EDTA with the use of Qiagen DNA Isolation Kit (Qiagen GmbH, Hilden, Germany).

The *VEGF* and *bFGF* alleles were detected using a polymerase chain reaction restriction fragment length polymorphism (PCR–RFLP) assay.

In brief, DNA was extracted from peripheral blood taken on EDTA using silica membranes (QiAmp Blood Kit, Qiagen, Hilden, Germany) following the recommendations of the manufacturer. A 208-bp-long fragment of the 3′ untranslated region (UTR) of the *VEGF* gene was amplified using the following primers: forward, 5′-GAG TGT CCC TGA CAA CAC TGG CA-3′, reverse, 5′-AGC AGC AGA TAA GGG ACT GGG GA-3′ as previously described [16]. The following primer pair was used for amplification of a 437-bp-long fragment of the *bFGF* gene promoter region: 5′-TGA GTT ATC CGA TGT CTG AAA TG-3′ and 5′-TAAC TTG AAT TAG ACG ACG CAG A-3′ [17]. The PCR cycling conditions were as follows: 94 °C for 3 min, followed by 30 cycles of: 94 °C for 30 s, 60 °C for 30 s, 72 °C for 30 s, with a final elongation step at 72 °C for 7 min. The PCR products were analyzed by electrophoresis in a 2 % agarose gel with ethidium bromide and visualized under UV. Then, the PCR products specific for the *VEGF* and *bFGF* gene were digested with *Nla*III and *Bse*IV restriction endonucleases (New England BioLabs^®^ Inc.), respectively, and analyzed on an agarose gel. Electrophoresis demonstrated the original 208-bp fragment (homozygous individuals for the *VEGF C* allele, lacking the *Nla*III site), three fragments of 208, 122 and 86 bp in length (heterozygous individual) or two fragments of 122 and 88 bp (homozygous individual for the *VEGF T* variant). For the *bFGF* polymorphism, the following electrophoresis patterns were observed: the original 437-bp fragment (homozygous individuals for the *bFGF C* allele, lacking the *Bse*IV site), three fragments of 437, 370 and 67 bp in length (heterozygous individual) or two fragments of 370 and 67 bp (homozygous individual for the *bFGF G* variant).

### Statistical analysis

Statistical evaluation was performed using Statistica 5.5 for Windows software. Genotype and allele frequencies were compared between the study groups by the Fisher’s exact or Person’s test. The Odds Ratio (OR) was calculated by Haldane’s modification of Woolf’s method, and the significance of its deviation from unity was estimated by Fisher’s exact test. Survival analyses were performed employing Kaplan–Maier analysis and log rank test. Probability values <0.05 were considered statistically significant, and those between 0.05 and 0.1 as indicative of a trend.

## Results and discussion

The former studies on the role of the VEGF and bFGF in B-CLL regarded their serum levels or cellular expression as prognostic markers of the progression of the disease [10–14].

As for the Polish patients with CLL, our former study documented the significantly higher bFGF levels in B-CLL patients than in controls [14], while Gora-Tybor et al. [13] described the differences in VEGF serum levels between patients and controls, and patients in Rai stage III and IV versus those in Rai stage 0–II (summarized in Table 1).

As VEGF production appeared to have a significant effect on the susceptibility to CLL and the course of the disease, in our present study, we wanted to determine whether functionally relevant polymorphism within the VEGF encoding gene (pp. 936 C > T in the 3′-UTR) could contribute to the risk of this malignant disease. The previous reports documented significantly lower VEGF plasma levels in carriers of the *936 T* allele what could be attributed to the 936 C/T exchange leading to the loss of a potential binding site for transcription factor AP-4 (activating enhancer-binding protein 4) [18].

The *bFGF (pp.* −*921 C* > *G)* promoter polymorphism was also studied. Polymorphisms within the promoter region of the *bFGF* gene may interfere with existing transcription factor binding sites or produce new binding sites and therefore influence the *bFGF* gene expression [17].

In the present study, C to T substitution at position 936 within the 3′-UTR of the *VEGF* gene and C to G substitution at position −921 within the promoter region of the *bFGF* gene were analyzed in order to determine whether the presence of these allelic variants is associated with susceptibility and progression of the disease in CLL patients.

As mentioned before, unfavorable CLL progression was reported to be associated with high VEGF levels and increased VEGF expression with a lack of the *VEGF 936 T* variant [18]. That is why the less frequent representation of the *VEGF T* allele among patients, especially those presented with more advanced disease, was expected. However, only a slight prevalence of the *VEGF T* variant was observed among patients as compared to healthy individuals (20/68 vs. 20/112, OR = 1.91, *p* = 0.095, Table 2). This relationship reached statistical significance when a group of high risk patients was considered. Among 11 patients in III or IV stage of the disease, 5 (45 %) were carrying the *T* allele as compared to 20 out of 112 (18 %) controls (OR = 3.81, *p* = 0.045, Table 2).

**Table 2:** Patients characteristics with respect to the distribution of the *VEGF T* and *bFGF G* variants in comparison with healthy population

CLL patients	*N*	*VEGF pp. 936*		*bFGF pp.* −*921*	
		*T*	*CC*	*G*	*CC*
		n (%)	n (%)	n (%)	n (%)
Sex, F/M					
Female	27	11	16	4	23
		31 %	59 %	15 %	85 %
Male	41	9	32	8	33
		21 %	78 %	19.5 %	80.5 %
Stage of the disease (Rai)					
Low risk					
0	17	6	11	4	13
		35 %	65 %	24 %	76 %
Intermediate					
I/II	28/12	9	31	6	34
		22.5 %	77.5 %	15 %	85 %
High risk					
III/IV	6/5	5^c^	6	2	9
		45.5 %	54.5 %	18 %	82 %
Serum β2-microglobulin^a^					
Normal	15	5	10	2	13
		23 %	67 %	13 %	87 %
Elevated	31	9	22	3	28
		29 %	71 %	10 %	90 %
Patients	68	20^b^	48	12	56
		29 %	71 %	18 %	82 %
Healthy individuals	112	20^b,^ ^c^	92	22	90
		18 %	82 %	20 %	80 %

The *VEGF T* variant was more frequently detected among patients presented with stage III/IV disease (high risk group). Individuals carrying the *VEGF T* variant are over three times more likely to develop high risk B-CLL

*CLL* chronic lymphocytic leukemia

^a^Elevated >1.80 mg/L, unknown for 22 cases

^b^(CLL patients vs. controls) OR = 1.91, *p* = 0.095

^c^(high risk patients vs. controls) OR = 3.82, *p* = 0.045

No other significant association was observed between the *VEGF* polymorphism and progression of the disease. The *VEGF* alleles and genotypes segregated similarly in patients with different stage of the disease according to Rai classification, beta2 microglobulin serum level and survival.

No other significant relationships were also observed for the *bFGF* polymorphism with either susceptibility to B-CLL (when compared to control group, Table 2) or progression of the disease.

Thus, no differences in *VEGF* and/or bFGF alelle and/or genotype distribution were noted between subgroups with stage 0–II versus III–IV according to modified Rai staging as well as males versus females (individual data not shown).

Intensive literature search was performed in order to compare the *VEGF* and *bFGF* alleles and genotypes distribution in Caucasian B-CLL patients and controls of other studies with our present results.

To our knowledge, there are no published data on the role of *VEGF* and *bFGF* polymorphisms in B-CLL (thus, our report present novel observations not previously described).

As for the *bFGF* gene polymorphism, similar distributions of *bFGF* alleles and genotypes in healthy Czech (*n* = 126) [17] and Slovenian (*n* = 107) [19], (*n* = 294) [20] were shown.

There were more studies investigating the distribution of the *VEGF* alleles showing comparable results. Allele frequencies of the present study are in agreement with those previously published for healthy Caucasians [21–23].

These data suggest that while the *bFGF (*−*921 C* > *G)* polymorphism does not significantly contribute to susceptibility and progression of the disease in Polish patients with B-CLL, the *VEGF (936 C* > *T)* polymorphism may be associated with high risk disease.

Obviously, these results should be regarded as preliminary and confirmed in the more extended study, including patients from the other centers.

## References

[1] Aguayo A, Kantarjian H, Manshouri T, Gidel C, Estey E, Thomas D, Koller C, Estrov Z, O’Brien S, Keating M, Freireich E, Albitar M (2000). Angiogenesis in acute and chronic leukemias and myelodysplastic syndromes. Blood.

[2] Shanafelt TD, Kay NE (2006). The clinical and biologic importance of neovascularization and angiogenic signaling pathways in chronic lymphocytic leukemia. Semin Oncol.

[3] Ghosh AK, Secreto CR, Knox TR, Ding W, Mukhopadhyay D, Kay NE (2010). Circulating microvesicles in B-cell chronic lymphocytic leukemia can stimulate marrow stromal cells: implications for disease progression. Blood.

[4] Chen H, Treweeke AT, West DC, Till KJ, Cawley JC, Zuzel M, Toh CH (2000). In vitro and in vivo production of vascular endothelial growth factor by chronic lymphocytic leukemia cells. Blood.

[5] Smolej L, Andrýs C, Maisnar V, Pour L, Malý J (2005). Plasma concentrations of vascular endothelial growth factor and basic fibroblast growth factor in lymphoproliferative disorders. Acta Medica (Hradec Kralove)..

[6] Frater JL, Kay NE, Goolsby CL, Crawford SE, Dewald GW, Peterson LC (2008). Dysregulated angiogenesis in B-chronic lymphocytic leukemia: morphologic, immunohistochemical, and flow cytometric evidence. Diagn Pathol..

[7] Farahani M, Treweeke AT, Toh CH, Till KJ, Harris RJ, Cawley JC, Zuzel M, Chen H (2005). Autocrine VEGF mediates the antiapoptotic effect of CD154 on CLL cells. Leukemia.

[8] Gehrke I, Gandhirajan RK, Poll-Wolbeck SJ, Hallek M, Kreuzer KA (2011). Bone marrow stromal cell-derived vascular endothelial growth factor (VEGF) rather than chronic lymphocytic leukemia (CLL) cell-derived VEGF is essential for the apoptotic resistance of cultured CLL cells. Mol Med.

[9] König A, Menzel T, Lynen S, Wrazel L, Rosén A, Al-Katib A, Raveche E, Gabrilove JL (1997). Basic fibroblast growth factor (bFGF) upregulates the expression of bcl-2 in B cell chronic lymphocytic leukemia cell lines resulting in delaying apoptosis. Leukemia.

[10] Menzel T, Rahman Z, Calleja E, White K, Wilson EL, Wieder R, Gabrilove J (1996). Elevated intracellular level of basic fibroblast growth factor correlates with stage of chronic lymphocytic leukemia and is associated with resistance to fludarabine. Blood.

[11] Molica S, Vitelli G, Levato D, Gandolfo GM, Liso V (1999). Increased serum levels of vascular endothelial growth factor predict risk of progression in early B-cell chronic lymphocytic leukaemia. Br J Haematol.

[12] Aguayo A, O’Brien S, Keating M, Manshouri T, Gidel C, Barlogie B, Beran M, Koller C, Kantarjian H, Albitar M (2000). Clinical relevance of intracellular vascular endothelial growth factor levels in B-cell chronic lymphocytic leukemia. Blood.

[13] Gora-Tybor J, Blonski JZ, Robak T (2005). Circulating vascular endothelial growth factor (VEGF) and its soluble receptors in patients with chronic lymphocytic leukemia. Eur Cytokine Netw.

[14] Wołowiec D, Dybko J, Wróbel T, Urbaniak-Kujda D, Jaźwiec B, Tomaszewska-Toporska B, Kapelko-Słowik K, Potoczek S, Kuliczkowski K (2006). Circulating sCD138 and some angiogenesis-involved cytokines help to anticipate the disease progression of early-stage B-cell chronic lymphocytic leukemia. Mediators Inflamm.

[15] Hallek M, Cheson BD, Catovsky D, Caligaris-Cappio F, Dighiero G, Dohner H, Hillmen P, Keating MJ, Montserrat E, Rai KR, Kipps TJ (2008). Guidelines for the diagnosis and treatment of chronic lymphocytic leukemia:a report from the International Workshop on Chronic Lymphocytic Leukemia updating the National Cancer Institute–Working Group 1996 guidelines. Blood.

[16] Papazoglou D, Galazios G, Papatheodorou K, Liberis V, Papanas N, Maltezos E, Maroulis GB (2005). Vascular endothelial growth factor gene polymorphisms and idiopathic recurrent pregnancy loss. Fertil Steril.

[17] Beránek M, Tschöplová S, Kanková K, Kuhrová V, Vácha J (2003). Genetic variation in the promoter region of the basic fibroblast growth factor gene. Hum Immunol.

[18] Renner W, Kotschan S, Hoffman C, Obermayer-Pietsch B, Pilger E (2000). A common 936C/T mutation in the gene for vascular endothelial growth factor is associated with vascular endothelial growth factor plasma levels. J Vasc Res.

[19] Kariz S, Grabar D, Krkovic M, Osredkar J, Petrovic D (2009). Polymorphisms in the promoter region of the basic fibroblast growth factor gene are not associated with myocardial infarction in a Slovene population with type 2 diabetes. J Int Med Res.

[20] Petrovic MG, Krkovic M, Osredkar J, Hawlina M, Petrovic D (2008). Polymorphisms in the promoter region of the basic fibroblast growth factor gene and proliferative diabetic retinopathy in Caucasians with type 2 diabetes. Clin Experiment Ophthalmol..

[21] Giacalone A, Montalto G, Giannitrapani L, Balasus D, Terranova A, Cervello M, Soresi M, Marasà L (2011). Association between single nucleotide polymorphisms in the cyclooxygenase-2, tumor necrosis factor-α, and vascular endothelial growth factor-A genes, and susceptibility to hepatocellular carcinoma. OMICS.

[22] Kämmerer PW, Toyoshima T, Eletr S, Kämmerer P, Kuhr K, Al-Nawas B, Brieger J (2010). Single nucleotide polymorphisms of the vascular endothelial growth factor gene associated with incidence of oral squamous cell carcinoma. J Oral Pathol Med.

[23] Rodrigues P, Furriol J, Tormo E, Ballester S, Lluch A, Eroles P (2012). The single-nucleotide polymorphisms +936 C/T VEGF and -710 C/T VEGFR1 are associated with breast cancer protection in a Spanish population. Breast Cancer Res Treat.

[24] Ferrajoli A, Manshouri T, Estrov Z, Keating MJ, O'Brien S, Lerner S, Beran M, Kantarjian HM, Freireich EJ, Albitar M. High levels of vascular endothelial growth factor receptor-2 correlate with shortened survival in chronic lymphocytic leukemia. Clin Can Res. 2001;7:795–9. 11309324

[25] Paesler J, Gehrke I, Gandhirajan RK, Filipovich A, Hertweck M, Erdfelder F, Uhrmacher S, Poll-Wolbeck SJ, Hallek M, Kreuzer KA (2010). The VEGF receptor tyrosine kinase inhibitors vatalanib and pazopanib potently induce apoptosis in chronic lymphocytic leukemia cells in vitro and in vivo. Clin Can Res..

